# Annexin A13 promotes tumor cell invasion *in vitro* and is associated with metastasis in human colorectal cancer

**DOI:** 10.18632/oncotarget.15523

**Published:** 2017-02-20

**Authors:** Guozhong Jiang, Pengju Wang, Weiwei Wang, Wencai Li, Liping Dai, Kuisheng Chen

**Affiliations:** ^1^ Department of Pathology, The First Affiliated Hospital of Zhengzhou University, Zhengzhou, 450052, China; ^2^ Sino-British Research Centre for Molecular Oncology, School of Basic Medical Sciences, Academy of Medical Sciences, Zhengzhou University, Zhengzhou, 450052, China; ^3^ Institute of Medical and Pharmaceutical Sciences in Zhengzhou University, Zhengzhou, 450052, China

**Keywords:** annexin A13, cell invasion, metastasis, colorectal cancer

## Abstract

**Purpose:**

Aberrantly upregulated expression of selected members of annexin, a group of calcium- and membrane-binding proteins, have been found to be associated with metastasis, poor prognosis, and other clinical characteristics in colorectal cancer (CRC), the third most diagnosed cancer. However, ANXA13 (encoding protein annexin A13), the original founder gene of the annexin A family, has not been studied carefully as a potential prognostic biomarker in CRC.

**Methods:**

The protein level of annexin A13 was determined by western blot in a panel of CRC cell lines. Tumor cell invasion was determined by a Matrigel *in vitro* invasion assay in selected CRC cells with either upregulated (via plasmid transfection) or downregulated (via siRNA treatment) expression of ANXA13. The clinicopathological features and prognostic values associated with ANXA13 expression were also evaluated in a group of 125 CRC patients.

**Results:**

ANXA13 was expressed at a high level in HCT116 and HT29 cells but undetected or at a lower level in SW620, SW48, and Rko cells. CRC cell invasion was promoted by ANXA13 overexpression in SW620 or Rko cells and was reduced by ANXA13 downregulation in HCT116 or HT29 cells. In CRC patients, ANXA13 expression levels correlated with lymph node metastasis and were associated with poor overall survival.

**Conclusions:**

ANXA13 is associated with CRC cell invasion *in vitro*, and with lymph node metastasis and poor survival in CRC patients. Our results indicate that ANXA13 can be exploited as a biomarker for its diagnostic and prognostic values.

## INTRODUCTION

Over 1.3 million patients are diagnosed with colorectal cancer (CRC) and nearly 0.7 million die from the disease each year, accounting for the third most diagnosed cancer and the fourth-leading cause of cancer death worldwide [1]. CRC is more prevalent in older populations with a median age of 70 years at diagnosis [1, 2]. Due to improvements in early diagnosis and personalized treatment, the 5 year survival rate has reached almost 65% in developed countries [3]. Currently, the most important prognostic factor is the stage at diagnosis. In the United States, the 5-year relative survival rate is 90.3% for patients with localized stage, 70.4% with regional spread, but only 12.5% with distant spread [4].

CRC is a set of heterogeneous diseases underlain by different molecular mechanisms that regulate tumorigenesis and are associated with different phenotypic presentations, prognostic values, and therapeutic responses [3]. Thus, molecules identified to be involved in these pathogenic processes are likely associated with tumor clinicopathological features, disease prognosis, and therapeutic response, and can be exploited as biomarkers. Such a group of molecules are annexins, a superfamily of calcium- and membrane-binding proteins that show altered expression pattern in various neoplasms [5, 6].

There are 12 annexin A subfamily members (A1-A11, and A13) in human that are involved in a range of cellular processes including membrane scaffolding, ion transportation, cell signaling, cell division, and apoptosis [7]. Family members including annexin A1, A2, A4, A5, A7, A10, and A11 have been suggested to be associated with specific clinicopathological characteristics, including metastatic status, in CRC and other cancers [8–16]. Surprisingly, ANXA13, which is highly expressed in the digestive system and believed to be the original founder gene of the annexin A family, has not been studied as a possible biomarker in CRC [17, 18]. Limited evidence suggests that ANXA13 may play a role in apoptotic cell-mediated immunosuppression [19], hypertension [20], and cell death recognition [21]. Interestingly ANXA13 is highly expressed in the human colon adenocarcinoma cell line HT29, suggesting that ANXA13 might be clinically relevant in CRC [17]. To determine its clinical value in CRC, we used a panel of CRC cell lines to investigate the expression level of ANXA13 in these cells and their potential roles in tumor development. In the present study, we evaluated the clinicopathological features and the diagnostic and prognostic values associated with ANXA13.

## RESULTS

### ANXA13 expression enhances invasion and migration in CRC cell lines

**Figure 1:**
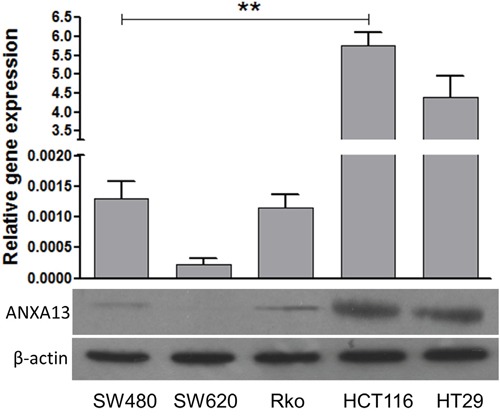
Western blot experiments show the expression of annexin A13 in CRC cell lines Western blot showing that annexin A13 (ANXA13) is expressed in selected CRC cell lines (Bottom). Summary graph showing that ANXA13 expression (normalized to β-actin) is significantly higher in HCT116 and HT29 cells than in SW620, Rko, and SW480 cells (top). Values are mean ± SEM; n = 3 each; *P < 0.05, **P < 0.01 by one-way ANOVA with Tukey's multiple comparisons test.

**Figure 2:**
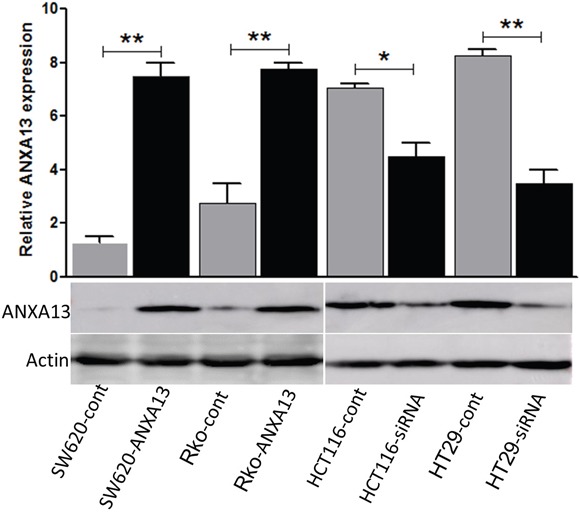
Annexin A13 level is upregulated by ANXA13 overexpression and downregulated by ANXA13-siRNA Western blot showing that annexin A13 (ANXA13) expression and β-actin control in SW620 and Rko cells transfected with plasmid vectors encoding control or ANXA13, or in HCT116 and HT29 cells treated with control or ANXA13 siRNA (bottom). Summary graph showing the level of ANXA13 in indicated CRC cells transfected with ANXA13-containing plasmid vectors or ANXA13-siRNA (top). Values are mean ± SEM; n = 3; *P < 0.05, **P < 0.01 by unpaired Student's t test.

**Figure 3:**
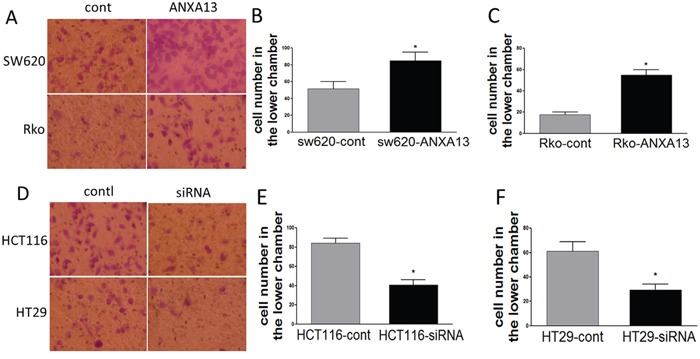
ANXA13 regulates CRC cell invasion *in vitro* invasion assays **(A)** Images showing invasive cells that migrated to the bottom chambers. **(B-C)** Summary graph showing that ANXA13-overexpression significantly increased the number of invasive cells in SW620 **(B)** and Rko **(C)** cells. **(D)** Images showing invasive cells that migrated to the bottom chambers. **(E-F)** Summary graph showing that ANXA13 siRNA significantly decreased the number of invasive cells in HCT116 **(E)** and HT29 **(F)** cells. Values are mean ± SEM; n = 3; *P < 0.05, **P < 0.01 by unpaired Student's t test.

**Figure 4:**
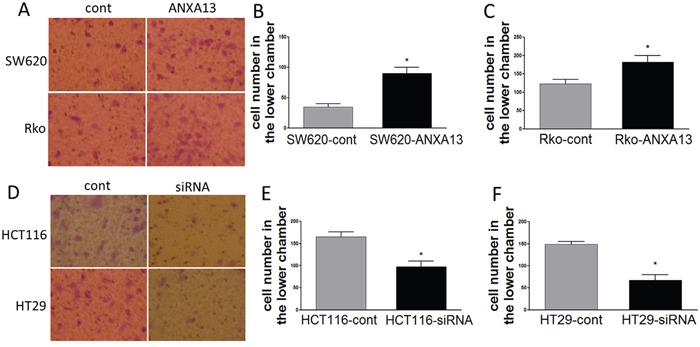
ANXA13 regulates CRC cell migration *in vitro* migration assays **(A)** Images showing cells that migrated through an uncoated filter to the bottom chamber. **(B-C)** Summary graph showing that ANXA13-overexpression enhanced cell migration in SW620 **(B)** and Rko **(C)** cells. **(D)** Images showing cells that migrated through an uncoated filter to the bottom chamber. **(E-F)** Summary graph showing that ANXA13 siRNA significantly decreased cell migration in HCT116 **(E)** and HT29 **(F)** cells. Values are mean ± SEM; n = 3; *P < 0.05, **P < 0.01 by unpaired Student's t test.

**Figure 5:**
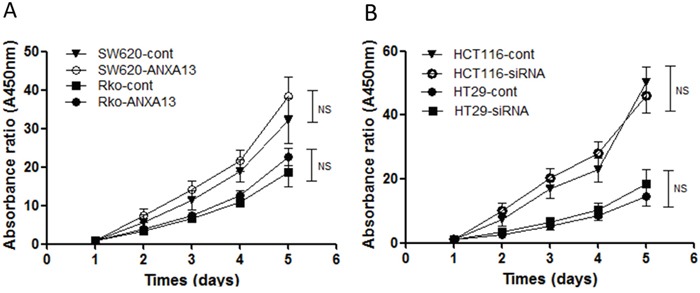
MTT assay indicates that ANXA13 has no effect on cell viability **(A)** No significant effect on cell viability measured by MTT was found in SW620 and Rko cells transfected with ANXA13. **(B)** No significant effect on cell viability was found in HCT116 and HT29 cells transfected by ANXA13-siRNA. Values are mean ± SEM; n = 3; NS, not significant by two-way ANOVA with Tukey's multiple comparisons test.

We first analyzed the relative expression levels of annexin A13 in a panel of CRC cell lines using western blotting. Annexin A13 expression was undetectable in SW620 cells, but expressed at low levels in SW480 and Rko cells, and at a high level in HCT116 and HT29 cells (Figure 1). To assess the functional significance of ANXA13 expression in CRC cells, we overexpressed ANXA13 in SW620 and Rko cells and downregulated ANXA13 in HCT116 and HT29 cells (Figure 2). For overexpression we transfected cells with pcDNA3.1-ANXA13 or pcDNA3.1 vector as control. For downregulation we treated cells with siRNA. ANXA13 was found significantly upregulated in ANXA13-overexpressing cells and downregulated in siRNA-exposed cells (Figure 2). We performed cell invasion and migration experiments to determine whether ANXA13 plays a role in these events. In cell invasion experiments using either 20% (as demonstrated in Figure 3) or 10% (data not shown) serum as a chemoattractant, we found that ANXA13 overexpression enhanced cell invasion (Figure 3A and 3C) whereas downregulation of ANXA13 expression significantly decreased cell invasion (Figure 3D and 3F). In the same fashion, cell migration was enhanced by ANXA13 overexpression (Figure 4A and 4C), but reduced by ANXA13 siRNA application (Figure 3D and 3F). Although resulting in a significant effect on tumor cell migration and invasion, ANXA13 overexpression showed no apparent effect on tumor cell proliferation as shown by MTT assays. We demonstrated that neither overexpression (Figure 5A) nor downregulation (Figure 5B) of ANXA13 had a significant effect on cell viability (determined by absorbance ratio at 450 nm) in SW620 or Rko cells.

### ANXA13 regulates MMP-9 expression through AKT-mediated phosphorylation

**Figure 6:**
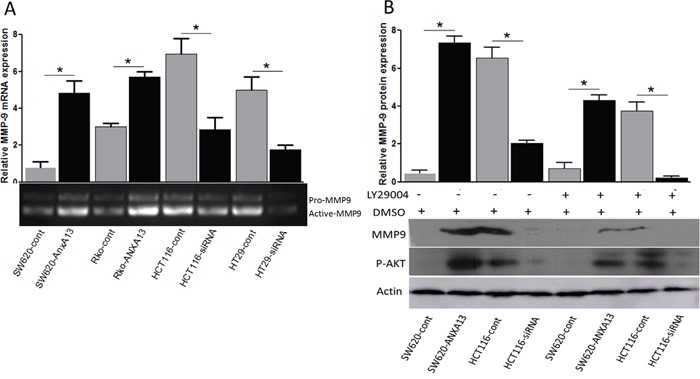
ANXA13 upregulates active MMP-9 through AKT phosphorylation **(A)** Zymographic assay showing that the level of active MMP-9 was upregulated by ANXA13 overexpression but downregulated by ANXA13-siRNA. **(B)** Western blot showing that ANXA13-mediated MMP-9 expression was abolished by AKT inhibitor LY29004, which inhibited AKT kinase function. Values are mean ± SEM; n = 3; *P < 0.05, **P < 0.01 by unpaired Student's t test.

To uncover the potential molecular pathways underlying ANXA13-enhanced tumor cell invasion and migration, we determined whether the ANXA13 downstream protein MMP-9, which is known for its role in cancer metastasis and tumor cell migration and invasion [24, 25], is involved in the process. Interestingly, we found that MMP-9 expression by Gelatin zymographic analysis was upregulated by ANXA13 overexpression in SW620 and Rko cells, but downregulated in HCT 116 and HT29 cells (Figure 6A). We further showed that this regulation was mediated through AKT phosphorylations. Western blotting demonstrated that ANXA13-mediated MMP-9 upregulation in SW620 cells was inhibited by the AKT inhibitor LY29004, which inhibits AKT's ability to phosphorylate its substrates (Figure 6B). In contrast, ANXA13 regulates endogenous MMP-9 expression in HCT116 cells, as shown by the downregulation of MMP-9 expression by ANXA13 siRNA (Figure 6B).

**Figure 7:**
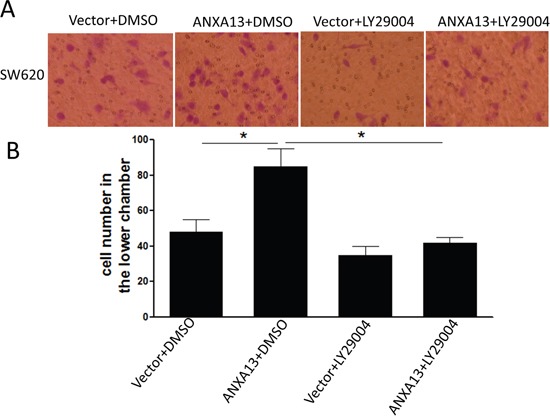
*in vitro* invasion assays show that ANXA13-enhanced tumor cell invasion is inhibited by AKT inhibitor LY29004 **(A)** Images showing invasive cells (SW620) that migrated to the bottom chamber. **(B)** Summary graph showing that ANXA13-overexpression significantly increased the number of invasive cells, which was abolished by LY29004 application. Values are mean ± SEM; n = 3; NS, not significant by two-way ANOVA with Tukey's multiple comparisons test.

To determine whether ANXA13-mediated AKT-phosphorylation and MMP-9 up-regulation play an important role in tumor cell invasion, we performed tumor cell invasion experiments in cultured SW620 cells overexpressing ANXA13 or control cells and treated with either the AKT inhibitor LY29004 or DMSO as a control (Figure 7). We found that the ANXA13-enhanced increase in the number of cells in the lower chamber (column 2 vs. 1: 85 ± 14.14 vs. 48 ± 9.90, P<0.05) was inhibited by LY29004 treatment (column 4 vs. 1: 42 ± 4.24 vs. 48 ± 9.90, P<0.55), suggesting that AKT activity plays a critical role in ANXA13-mediated tumor cell invasion.

### ANXA13 expression is positively correlated with metastasis and predicts the prognosis for CRC

**Figure 8:**
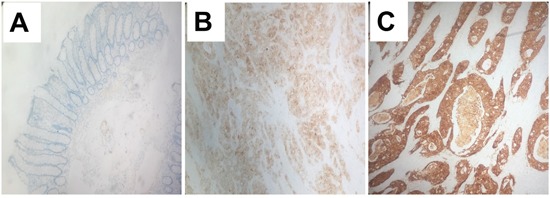
Expression of annexin A13 (ANXA13) in human CRC Immunohistochemistry images (400 X) showing the expression of annexin A13 (ANXA13) in human control or CRC samples. ANXA13 is undetectable in normal epithelium **(A)**, but expressed at a moderate **(B)** or high **(C)** level in CRC tissues.

**Figure 9:**
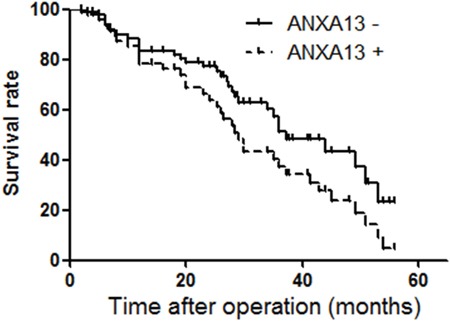
ANXA13 expression and survival of patients with CRC Survival analysis of CRC with or without expression of ANXA13 in tumor tissues was performed by Kaplan-Meier survival analysis. Cox repression test showed p<0.05 (*p*=0.003).

To elucidate whether ANXA13 overexpression is associated with tumor invasion and metastasis in human patients, we investigated annexin A13 expression in human CRC tissues and its relationship with clinicopathological factors. We observed that annexin A13 was not expressed in normal epithelial cells, but expressed at moderate or high level in tumor cells in 58.4% (73/125) of CRC cases (Figure 8 and Table 1). Upon close examination, annexin A13 was expressed mainly in the cytoplasm of cancer cells (Figure 8). We found that annexin A13 expression was not correlated with tumor stage, tumor differentiation, gender or age (Table 1). However, there was a significant association between annexin A13 expression and lymph node metastases (*p*=0.003). In this study, the followups for 92 of the 125 cancer patients were available for OS analysis. The median survival time in the CRC patient groups without annexin A13 expression (37.7 months) was significantly longer (*p*=0.003) than for the groups with annexin A13 expression (29 months, Figure 9). Moreover, multivariate Cox regression analysis (Table 2) indicated that both annexin A13 expression and lymph metastases were significantly associated with patient survival (*p*=0.028 and 0.005, respectively). These results suggest that annexin A13 expression correlated with the metastatic status in human CRC and could be further exploited as a useful biomarker to predict the prognosis of human CRC.

**Table 1:** Clinicopathological characteristics of 125 patients with colorectal cancer

	Annexin A13 expression		
	-n=52(41.3%)	+n=73(58.7%)	*p* value*
Age			
<50	11(8.8%)	14(11.2%)	0.786
≧50	41(32.8%)	59(47.2%)	
Gender			
Female	25(20.0%)	37(29.6%)	0.774
Male	27(21.6%)	36(28.8%)	
TNM stage			
I	12(9.6%)	8(6.4%)	0.246
II	13(10.4%)	23(18.4%)	
III	25(20.0%)	36(28.8%)	
IV	2(1.6%)	6(4.8%)	
Differentiation			
Poorly	2(1.6%)	9(7.2%)	0.1656
Moderately	37(29.6%)	52(41.6%)	
Well	13 (11.2%)	12(9.6%)	
Node metastasis			
Positive	21(16.8%)	49(39.2%)	0.003**
Negative	31(24.8%)	24(19.2%)	

^*^Χ^2^ test, **P<0.01.

**Table 2:** Multivariate analysis of the factors correlated with survival in human colorectal cancer

Variable	Hazard ratio	95.0% CI	p Value
Annexin A13 expression	2.311	1.309 to 4.038	0.028*
Lymph node metastasis	5.831	1.581 to 10.745	0.005**
Tumor differentiation	0.652	0.634 to 3.234	0.733
TNM	1.242	0.836 to 1.462	0.511
Gender	1.231	0.984 to 3.847	0.621
Age	0.732	0.463 to 2.322	0.921

Multivariate analysis of the factors correlated with survival by Cox regression

*p<0.05,**p<0.01

## DISCUSSION

Annexins are capable of binding negatively charged phospholipids in a calcium-dependent manner and are involved in a variety of cellular processes including vesicle transport, membrane scaffolding, calcium signaling, cell growth, cell division, and apoptosis [7]. Their expression levels, which are tissue-specific under normal conditions, are consistently altered in a wide range of neoplasms [6]. Clinical evidence has shown that several annexin A subfamily members are associated with specific types of clinicopathological features and have prognostic values [8–16]. While some members have been extensively studied, others, including ANXA13, have not received enough or any attention at all in CRC. In this study, the potential role of ANXA13 in CRC carcinogenesis and its clinicopathological and prognostic relevance were investigated for the first time in both *in vitro* CRC cells and in human patients.

Determining lymph node metastatic status is critical for correct tumor classification and proper treatment selection in CRC. Mechanistic studies suggested that selected members of annexins are important in tumor invasion and metastasis [5]. Clinical evidence also showed that several members of annexins are associated with lymph node metastasis and tumor cell invasiveness. A recent study found that the membrane expression pattern of Annexin A2 was associated with high invasiveness and lymph node metastasis [10]. Similarly, another study has shown that increased expression of annexin A2 correlated with poor differentiation, late stage, and lymph node metastasis [11]. In addition to A2, annexin A5 was another member found to be associated with (liver) metastasis in CRC [13]. Interestingly, annexin A1 has been identified as one of the early metastasis-associated proteins in sentinel lymph node micrometastasis (SLNMM) of CRC through comparative proteomic analysis [12]. In the current study, we have shown that annexin A13 expression was positively correlated with lymph node metastasis in 125 patients with CRC. Moreover, our *in vitro* study using CRC cell lines indicated that CRC tumor cell invasion was enhanced by ANXA13 overexpression in SW620 cells but decreased by ANXA13 siRNA-mediated downregulation in HCT116 cells. Meanwhile, ANXA13 overexpression had no effect on the proliferation of SW620 cells, suggesting the functional specificity of ANXA13-mediated pathogenic processes in CRC. These results together with previously published studies [8–16] suggest that annexin members A13, A2, A5, and A1 are clinically relevant in CRC metastasis.

Although associated with CRC metastasis, ANXA13 expression in this cohort of patients was not associated with their TMN stage, which has been suggested as one of the most validated prognostic factors in CRC within East Asian patients [26]. Surprisingly, TMN stage in this cohort of patients was also not significantly associated with OS. In addition, this cohort of patients also showed shorter median OS. These results suggest that the clinical manifestations of this patient population, including that about half of the patients were diagnosed at stage III, differentiate it from other studied cohorts of CRC patients. Such factors may contribute in part to the shorter OS, and that only 8 patients accounting for 6.4% of the total population were diagnosed at stage IV, which may mask the association between TNM stage and OS or ANXA13 expression. The fact that only sporadic adenocarcinomas were included in the study may also be a contributing factor to the lack of association between TNM stage and OS.

Our *in vitro* experiments in cultured CRC cells strongly suggest that ANXA13-mediated the expression of the active form of MMP-9, which is known for its role in tumor metastasis [22]. These studies also demonstrated that ANXA13-enhanced MMP-9 expression was regulated through AKT phosphorylation. Importantly, our results suggest that ANXA13 enhances tumor cell invasion through activation of the AKT-MMP-9 pathway, since the AKT inhibitor LY29004 abolished ANXA13-enhanced tumor cell invasion. These results are consistent with previously published studies showing that AKT/MMP-9 activation is required for tumor cell invasion and metastasis [27, 28]. Therefore, it is possible that ANXA13-mediated activation of AKT and MMP-9 plays a pivotal role in tumor cell invasion and metastasis in CRC.

Our study and others’ have suggested that annexins can be exploited as biomarkers for their prognostic values. In this study, we demonstrated that the expression of annexin A13 was associated with poor prognosis in CRC patients. Similarly, others have shown that annexin A2 expression can also serve as a predicting factor for both OS and disease recurrence [11]. Annexin A5 upregulation was found to be associated with advanced tumor stage, increased recurrence and lower OS in CRC [13]. Interestingly, the expression of annexin A3 correlated not only with poor prognosis but also the expression of hypoxia-inducible factor-1α (HIF-1α), an important molecule in the angiogenesis of tumors [29], suggesting a role for annexins in angiogenesis to promote metastasis. Intriguingly, annexin members may have contradictory roles and prognostic values in different types of cancers. For example, the upregulation of annexin A4 as well as A7 promotes tumor progression and is associated with poor prognosis and drug resistance in multiple types of cancers including CRC, but function as tumor suppressors in other types of cancers [8, 9]. It is not clear whether annexin A13 has an opposite function in different types of cancers.

Studies have suggested that altered annexin expression is also associated with genetic abnormalities and altered gene expression. Cancers with the CpG island methylator phenotype (CIMP) represent a clinically and etiologically distinct group characterized by epigenetic instability [30]. Annexin A10 has been found to be strongly associated with CIMP, microsatellite instability, and BRAF mutation, and thus can be exploited as a biomarker for the serrated pathway in invasive CRCs with poor prognosis [14, 15]. Increased expression of annexin A1 has been shown to be correlated with KRAS mutation, suggesting that annexin A1 is specifically involved in KRAS mutation-mediated tumor development or metastasis in CRC [31]. It is worth noting that the upregulation of annexin A1 is associated with increased levels of carcinoembryonic antigen (CEA), which is only produced in gastrointestinal tissue during fetal development and is one of the most commonly used markers in colorectal cancer [32]. Whether ANXA13 and other members of this protein family are associated with these genetic abnormalities are the subjects for future studies.

In conclusion, we have shown that ANXA13 was aberrantly upregulated in cultured CRC cells. ANXA13 overexpression promoted CRC cell invasion *in vitro*, suggesting a role in CRC cell metastasis. In human patients, ANXA13 upregulation is associated with lymph node metastasis and poor survival. Therefore, ANXA13 can be exploited as a biomarker for its diagnostic and prognostic values.

## MATERIALS AND METHODS

### Patients

A total of 125 patients with primary sporadic adenocarcinomas were enrolled in this study. The patients were admitted into the First Affiliated Hospital of Zhengzhou University, Zhengzhou, Henan, China between 2000 and 2001. Written consent was obtained from all participants for sample collection and participation in this study, which was approved by our institutional ethics committee. No preoperative chemotherapy, radiotherapy or other therapies were performed in this cohort of patients. Samples were preserved and stored as formalin-fixed, paraffin-embedded (FFPE) tissue blocks. Among 125 patients, 92 patients were qualified for survival analysis, including those who died within the 5-year follow-up period or were still alive at the end of the follow-up. All other patients were excluded from this analysis.

### Cell culture and DNA transfection

The human colorectal cancer cell lines SW620, SW480, Rko, HCT116, and HT29 cells were obtained from the Type Culture Collection of the Chinese Academy of Sciences, Shanghai, China. These cells were cultured in Dulbecco's modified Eagle's medium (DMEM) supplemented with 10% fetal bovine serum (FBS), 50 mg/ml streptomycin, and 50 mg/ml penicillin at 37°C with 5% CO_2_. To overexpress ANXA13, SW620 and Rko cells were transfected with pcDNA3.1-ANXA13 or pcDNA3.1 vector as control using Effectene reagent (Qiagen, USA) per manufacturer's instruction. To downregulate ANXA13, ANXA13 siRNA (SMARTpool 10 nM, Dharmacon, USA) or negative control siRNA (siNTC 10 nM, Dharmacon) were used to transfect HCT116 and HT29 cells using DharmaFECT transfection reagent (Dharmacon) per vender's instruction. The cells were allowed to grow for 48 h after transfection for both overexpression and downregulation experiments. The specificity of siRNAs was confirmed by western blot showing that ANXA13 had no or negligent downregulation in HCT116 cells expressing other ANXA13 members including A1, A2, A4, A5, A7, A10, and A11 (data not shown). Akt activity was inhibited by applying LY29004 (Calbiochem, USA) in cultured SW620 or HCT116 cells.

### Western blotting

The following antibodies were used in the western blot experiments: ANXA13, rabbit polyclonal (ab105802, Abcam, USA; the specificity of this antibody was further verify by a second ANXA13 antibody: goat polyclonal, AF4149-SP, R&D System, USA); β-actin, mouse monoclonal (clone AC-74, A2228, Sigma); MMP9, rabbit polyclonal (ab38898, Abcam); Phospho-Akt (Thr308) (244F9), Rabbit mAb #4056 (Cell Signaling Technology). Protein lysates were prepared from cultured cells in ice-cold Tris buffer (20 mmol/L; pH 7.5) containing NaCl (137 mmol/L), EDTA (2 mmol/L), Triton X (1%), glycerol (10%), NaF (50 mmol/L), DTT (1 mmol/L), and a protease inhibitor cocktail (P8340, Sigma, USA). After electrophoresis (SDS-PAGE), protein samples were transferred onto Hybond-P membranes (Amersham Biosciences). The blot was blocked in TBS with 5% nonfat milk and 0.1% Tween 20, followed by incubation with primary antibody (RT 1h) and HRP-tagged goat anti-rabbit or -mouse IgG secondary antibody (sc-2030 or sc-2357, Santa Cruz, USA). The blots were visualized and quantitated using Amersham ECL Western Blotting Detection Kit (Amersham Biosciences).

### Gelatin zymography assay

The active MMP-9 in the supernatant of cultured SW620, Rko, HCT116, and HT29 were detected and quantitated as described in a previously published study [22]. In brief, the abovementioned cells were seeded into different wells in a 24-well plate format and cultured at 37°C for 24 h. The conditioned medium was collected by centrifugation with 3000 rpm for 10 min at 4°C. MMP-9 and its active type contained in the conditioned medium were detected by Gelatin zymographic analysis and quantitated according to the method described in a previously published study by Pei et al. [22].

### Immunohistochemistry

The expression of annexin A13 was examined by immunohistochemistry on FFPE sections using a rabbit polyclonal antibody against annexin A13 (ab105802, Abcam, USA). Annexin A13 expression was evaluated in both tumor and adjacent normal tissues. Tissue sections were dewaxed, rehydrated, and followed by heat-induced antigen retrieval. Endogenous peroxidase activity was removed by applying 3% H_2_O_2_ for 5 minutes. After blocking with 5% normal goat serum, primary antibody was applied overnight at 4°C, followed by HRP-tagged goat anti-rabbit IgG secondary antibody (sc-2030, Santa Cruz, USA). Immunosignals were then visualized using diaminobenzidine and slightly counterstained with hematoxylin. Sections were evaluated for intensity under light microscope (no staining = 0; light staining = 1; moderate = 2; strong = 3). Any IHC intensity greater than 0 was defined as IHC positive.

### MTT assay

In cultured cells, 10 μL of 5 mg/mL MTT [3-(4, 5-dimethylthiazol-2-yl)-2, 5-diphenyltetrazolium bromide] (Sigma, USA) in PBS was added (final concentration 0.5 mg/mL) and incubated for 4 h. The MTT solution and medium were removed and 100 μL DMSO was added to each well. Absorbance was measured at 450 nm using the ELISA microplate reader.

### *in vitro* invasion and migration assays

CRC cell invasion and migration assays were performed as described in a published study elsewhere with minor modification [23]. In brief, CRC cells (2-3×10^5^) were plated on Matrigel-coated or uncoated upper chambers (24-well transwell insert with 8.0 μm pore size, BD Bioscience, USA) for migration or invasion assays. Two hours after seeding, the medium in the upper chambers was aspirated and replaced with serum-free medium, while medium in the lower chambers containing 20% or 10% serum served as a chemoattractant. Cells were incubated for 24 h and then fixed. Cells that migrated to the bottom chambers were photographed and counted.

### Statistical analysis

Statistical analyses were conducted using SPSS 16.0 software (Alteryx, USA). Data were presented as mean ± SEM. Statistical significance was determined by Student's *t*-test for pairwise comparisons and ANOVA for multiple comparisons. Multivariate Cox regression was used for survival analysis. P < 0.05 was considered statistically significant.
