# Correction: Annexin A13 promotes tumor cell invasion *in vitro* and is associated with metastasis in human colorectal cancer

**DOI:** 10.18632/oncotarget.28247

**Published:** 2023-03-21

**Authors:** Guozhong Jiang, Pengju Wang, Weiwei Wang, Wencai Li, Liping Dai, Kuisheng Chen

**Affiliations:** ^1^Department of Pathology, The First Affiliated Hospital of Zhengzhou University, Zhengzhou 450052, China; ^2^Sino-British Research Centre for Molecular Oncology, School of Basic Medical Sciences, Academy of Medical Sciences, Zhengzhou University, Zhengzhou 450052, China; ^3^Institute of Medical and Pharmaceutical Sciences in Zhengzhou University, Zhengzhou 450052, China; ^*^These authors have contributed equally to this work

**This article has been corrected:** In Figure 3, the images of control cells SW620 (3A, upper left panel) and HTC116 (3D, upper left panel) were accidentally switched with each other. The corrected Figure 3, produced using the original data, is shown below. The authors declare that these corrections do not change the results or conclusions of this paper.

Original article: Oncotarget. 2017; 8:21663–21673. 21663-21673. https://doi.org/10.18632/oncotarget.15523

**Figure 3:**
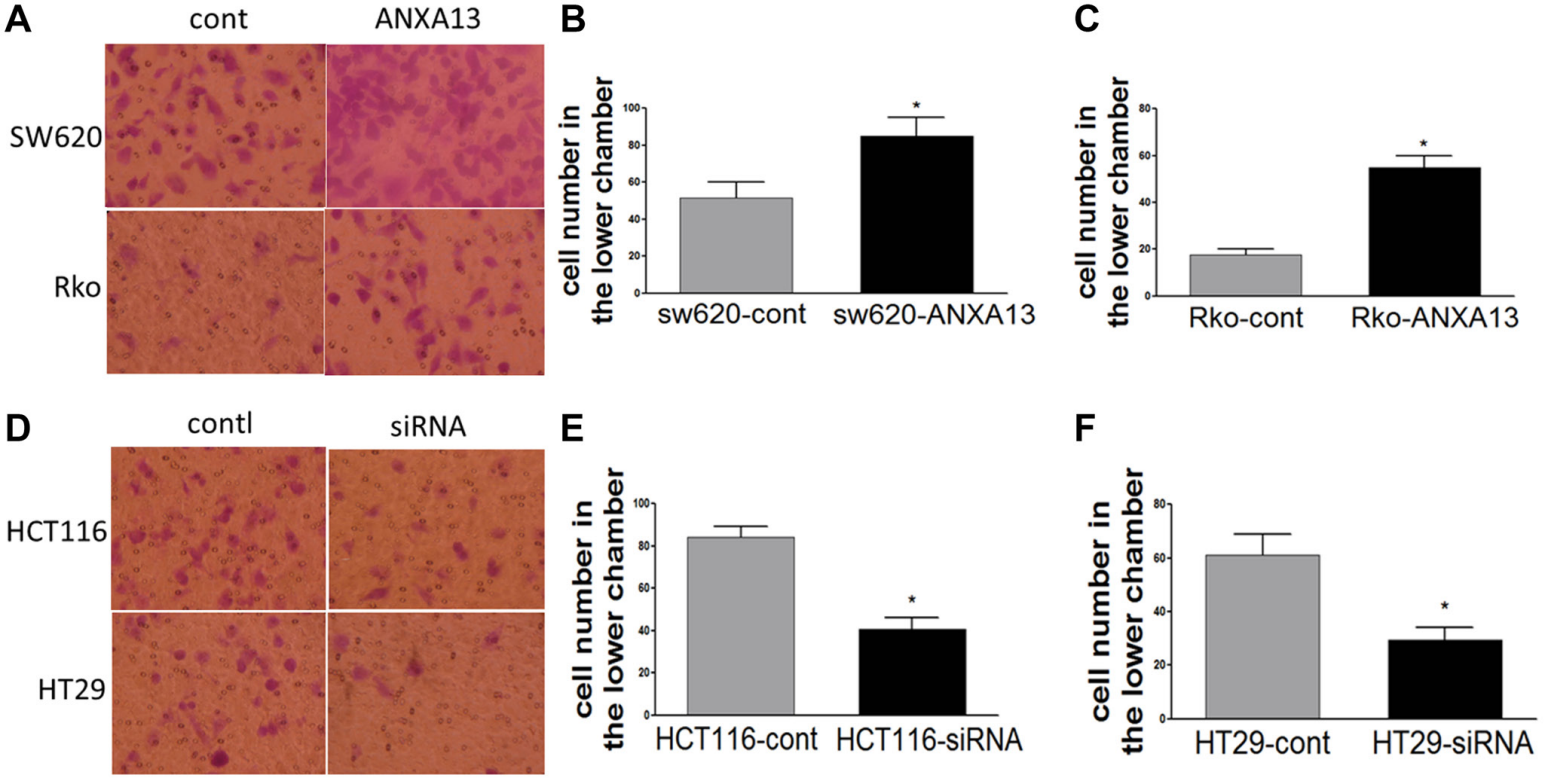
ANXA13 regulates CRC cell invasion *in vitro* invasion assays. (**A**) Images showing invasive cells that migrated to the bottom chambers. (**B**, **C**) Summary graph showing that ANXA13-overexpression significantly increased the number of invasive cells in SW620 (B) and Rko (C) cells. (**D**) Images showing invasive cells that migrated to the bottom chambers. (**E**, **F**) Summary graph showing that ANXA13 siRNA significantly decreased the number of invasive cells in HCT116 (E) and HT29 (F) cells. Values are mean ± SEM; *n* = 3; ^*^ *P* < 0.05, ^**^ *P* < 0.01 by unpaired Student’s *t* test.

